# Electronic Toys Decrease the Quantity and Lexical Diversity of Spoken Language Produced by Children With Autism Spectrum Disorder and Age-Matched Children With Typical Development

**DOI:** 10.3389/fpsyg.2022.929589

**Published:** 2022-07-01

**Authors:** Courtney E. Venker, Jennifer R. Johnson

**Affiliations:** Lingo Lab, Department of Communicative Sciences and Disorders, Michigan State University, East Lansing, MI, United States

**Keywords:** autism (ASD), play, toys and games industry, language, intervention

## Abstract

Many young children with autism spectrum disorder (ASD) have language delays. Play-based interactions present a rich, naturalistic context for supporting language and communication development, but electronic toys may compromise the quality of play interactions. This study examined how electronic toys impact the quantity and lexical diversity of spoken language produced by children with ASD and age-matched children with typical development (TD), compared to traditional toys without electronic features. Twenty-eight parent-child dyads (14 per group) played with both electronic and traditional toy sets in a counter-balanced order. We transcribed child speech during both play sessions and derived the number of utterances and number of different word (NDW) roots per minute that children produced. Children with ASD and children with TD talked significantly less and produced significantly fewer unique words during electronic toy play than traditional toy play. In this way, children appear to take a “backseat” to electronic toys, decreasing their communicative contributions to play-based social interactions with their parents. These findings highlight the importance of understanding how toy type can affect parent-child play interactions and the subsequent learning opportunities that may be created. Play-based interventions for children with ASD may be most effective when they incorporate traditional toys, rather than electronic toys.

## Introduction

Autism spectrum disorder (ASD) is a neurodevelopmental disorder characterized by social communication impairments, repetitive behaviors, and restricted interests that currently affects 1 in 44 children in the United States (American Psychiatric Association, 2013; Maenner et al., 2021). Impairments in structural language skills (i.e., vocabulary and grammar) are not required for a diagnosis of ASD (American Psychiatric Association, 2013). Nonetheless, many young children with ASD demonstrate severe delays in language development—lagging far behind their peers with typical development (TD) in both receptive and expressive (i.e., spoken) language skills (Charman et al., 2003; Tager-Flusberg et al., 2005; Ellis Weismer and Kover, 2015).

Many intervention approaches have been developed to address early language and communication delays in children with ASD. A growing number of autism interventions promote the creation of naturalistic, play-based interactions to facilitate children’s language and communication development (Dawson et al., 2010; Sussman, 2012; Schreibman et al., 2015; Binns and Oram Cardy, 2019; Bruinsma et al., 2020; Rogers et al., 2020). Within these interventions, parents and caregivers are commonly taught to create play interactions that limit distractions and prioritize social communicative exchanges. In line with social interactionist and transactional theories of language development, the rationale is that play provides a developmentally appropriate social context for language learning that maximizes children’s engagement and motivation and increases the likelihood that new skills will generalize to everyday settings (Sameroff and Fiese, 2000; Camarata and Yoder, 2002; Schreibman et al., 2015; Bruinsma et al., 2020; Bottema-Beutel and Kim, 2021).

Although play interactions have the potential to serve as an effective learning context for children with ASD, different types of toys may affect the quality of parent-child play interactions and the learning opportunities they provide (O’Brien and Nagle, 1987; Levin and Rosenquest, 2001; Miller et al., 2017). In recent years, electronic toys—toys that talk, sing, play music, and/or have flashing lights—have become increasingly common relative to traditional toys without technological features (Levin and Rosenquest, 2001; Radesky and Christakis, 2016). Contrary to marketing claims that electronic toys offer educational and developmental benefits (Levin and Rosenquest, 2001; Healey et al., 2019; Hassinger-Das et al., 2021; Zero to Three, 2021), numerous studies have shown that electronic toys decrease parent spoken language and responsiveness, compared to traditional toys (Wooldridge and Shapka, 2012; Zosh et al., 2015; Sosa, 2016; Miller et al., 2017; but see Sung, 2018).

Wooldridge and Shapka (2012) conducted an in-home study of parents playing with their typically developing young children (16–24 months old). Relative to traditional toys, electronic toys decreased the quality of parent behaviors associated with responsiveness and teaching. In a study of 10- to 16-month-old infants with TD, Sosa (2016) found that electronic toys were associated with fewer parent words, parent responses, and conversational turns, compared to traditional toys. Similarly, Zosh et al. (2015) found that parents of 24-month-old children with TD who played with electronic toys produced a significantly lower proportion of unique words than parents who played with traditional toys. Overall, these findings suggest that “parents tend to let the toys do the talking for them” (Sosa, 2016, p. 136) when playing with electronic toys, which may have detrimental effects on children’s language development (also see Wooldridge and Shapka, 2012; Miller et al., 2017).

Though most research on electronic toys has focused on parents of children with TD, we recently conducted the first published study (Sturman et al., 2022) investigating how electronic toys affect play interactions between children with ASD (2–4 years old) and their parents, compared to traditional toys. We also included a group of children with TD of the same chronological age. Consistent with findings in TD, parents in both groups talked significantly less and produced a significantly fewer unique vocabulary words when playing with electronic toys than traditional toys. Electronic toys also elicited significantly more pause time than traditional toys. Overall, these findings closely align with prior research in suggesting that electronic toys reduce the quality and quantity of parent language input provided to young children.

Understanding the impact of electronic toys on parent spoken language is important, given robust evidence that child language outcomes are closely linked with the quality and quantity of parent language input they receive (Hart and Risley, 1995; Hoff and Naigles, 2002; Huttenlocher et al., 2010; Rowe, 2012; Adamson et al., 2020). However, our recent findings (Sturman et al., 2022) raise an important question: how do electronic toys affect *children’s* spoken language, relative to traditional toys? Are differences in parent spoken language paralleled by differences in the spoken language produced by children with ASD or children with TD? Prior studies of children with TD have not investigated the impact of electronic toys on children’s spoken language—likely because of the young age of their participants. However, there is evidence that infants with TD produce fewer directed vocalizations and gestures when playing with electronic toys than traditional toys (Miller et al., 2017; also see Sosa, 2016).

The goal of the current study was to determine how toy type (traditional vs. electronic) affects the quantity and lexical diversity of spoken language produced by children with ASD and age-matched children with TD (2–5 years old). Based on prior studies (Wooldridge and Shapka, 2012; Zosh et al., 2015; Sosa, 2016; Miller et al., 2017) and on our findings regarding parent spoken language, we hypothesized that the quantity and lexical diversity of spoken language would be significantly lower during electronic than traditional toy play in both groups.

## Materials and Methods

### General Procedure

The study was approved by the Institutional Review Board at Michigan State University as part of a larger research project focused on language and visual attention in children with ASD (R21 DC 016102; Venker, PI). All parents provided written informed consent before participating. Parent-child dyads visited the lab on two separate days. They completed several activities related to language development, including standardized assessments and parent-child play sessions (described below).

### Participants

Twenty-eight parent-child dyads participated (*n* = 14 with ASD, *n* = 14 with TD). Families were recruited through a university email listserv for parents and caregivers, flyers posted in the community, and word of mouth. All children in the ASD group had previously been diagnosed with ASD, per parent report. The Autism Diagnostic Observation Schedule, Second Edition (ADOS-2; Lord et al., 2012a,b) was administered by a research-reliable examiner to confirm children’s existing ASD diagnoses. Module selection was based on age and language level, as described in the ADOS-2 manual. Two children received the Toddler Module (for children 12–30 months old), five children received Module 1: Few to no words, three children received Module 1: Some words, two children received Module 2: Younger than 5, and two children received Module 2: 5 or older. The ADOS-2 also provided calibrated severity scores, which indicate overall autism severity.

Parents reported no developmental concerns for children in the TD group. All families of children with TD completed the Lifetime Form of the Social Communication Questionnaire (Rutter et al., 2003) and scored at or below the cutoff score of 15, which indicated no need for further ASD evaluation.

There were 14 mothers in the ASD group, and 11 mothers and 3 fathers in the TD group. Children in the ASD group (11 male, 3 female; 93% Caucasian, 7% Black or African American; 100% non-Hispanic) and children in the TD group (5 male, 9 female; 93% Caucasian, 7% more than one race; 14% Hispanic, 86% non-Hispanic) were 2–5 years old. A Wilcoxon signed-rank test revealed no significant difference in the mean age of the children with ASD and the children with TD (*p* = 0.529). A Fisher’s Exact Test revealed that the proportion of males vs. females in the ASD and TD groups did not significantly differ (*p* = 0.054).

To assess receptive and expressive language abilities, we administered the Auditory Comprehension and Expressive Communication Scales of Pre-school-Language Scales, 5th edition (PLS-5; Zimmerman et al., 2011) to all participants. The PLS-5 provides an in-depth characterization of receptive and expressive language abilities, including vocabulary, grammar, literacy, and narrative skills. We assessed visual organization, memory, sequencing, and spatial awareness using the Visual Reception scale from the Mullen Scales of Early Learning (Mullen, 1995). The children with ASD had significantly lower scores than the children with TD on both the PLS-5 and the Mullen, indicating weaker language and cognitive skills (a topic we return to in the section “Discussion”; see Table 1). The number of children with ASD who scored 1.5 *SD* or more below the mean on the PLS-5 was 9/14 for the Expressive Communication Scale and 10/14 for the Auditory Comprehension Scale. Similarly, 10/14 children with ASD scored 1.5 *SD* or more below the mean on the Mullen Visual Reception Scale. In contrast, no child with TD scored more than 1 *SD* below the mean for either measure, indicating language and cognitive skills within the average range.

**TABLE 1:** Child demographic information.

	ASD group	TD group
		
	*Mean (SD)*	*Mean (SD)*
	range	range
Chronological age (months)	43.5 (12.86)	46 (14.45)
	26–71	25–67
PLS-5 AC standard score	63.71 (16.37)	116.71 (8.54)
	50–98	106–130
PLS-5 AC percentile	6.79 (12.56)	83.5 (11.97)
	1–45	66–98
PLS-5 AC age equivalent (in months)	23.57 (14.97)	58.21 (20.99)
	13–60	31–95
PLS-5 EC standard score	70.43 (13.02)	118.79 (16.15)
	50–93	96–148
PLS-5 EC percentile	6.85 (9.50)	80.21 (20.95)
	1–32	39–99
PLS-5 EC age equivalent (in months)	24.43 (13.09)	58.07 (19.20)
	9–59	33–95
Mullen VR T-score	27.71 (9.13)	61.86 (10.63)
	20–46	42–80
Mullen VR age equivalent (in months)	29.00 (15.42)	53.29 (12.54)
	14–66	27–69
ADOS-2 severity score	8.71 (1.33)	–
	6–10	

*ASD, autism spectrum disorder; TD, typical development.*

*Groups differed significantly at p < 0.001 on all variables except chronological age (p = 0.633). PLS-5, pre-school language scale, 5th Edition; AC, auditory comprehension scale; EC, expressive communication scale; PLS-5, standard scores have a mean of 100 and SD of 15. Mullen VR = mullen scales of early learning, visual reception scale. Mullen t-scores have a mean of 50 and SD of 10. ADOS-2 severity scores = autism diagnostic observation schedule, 2nd Edition, calibrated severity score. Calibrated severity scores range from 1 to 10, with 1–2 indicating minimal-to-no evidence of autism spectrum-related symptoms, 3–4 indicating low evidence, 5–7 indicating moderate evidence, and 8–10 indicating a high level of autism spectrum-related symptoms.*

### Parent-Child Play Sessions

Parent-child dyads engaged in two, one-on-one play sessions in the lab for 10-min periods, with each session occurring on a different day. Play sessions took place in the laboratory setting, in a quiet room equipped with a table and chairs and a set of toys placed on the floor. Each dyad had the room to themselves. Parent were asked to play with their child as they normally would at home with the set of toys provided. Sessions were recorded using cameras placed around the room and an overhead microphone.

Each parent-child dyad played with the traditional toy set on 1 day and the electronic toy set on the other. The items in the toy sets were closely matched. Each set included a barn with animals, a shape sorter, spiky sensory balls, three vehicles, a puzzle, and a pull toy dog (see Table 2). The toys in the electronic toy set talked, sang, made music, and/or flashed lights. Each toy in the electronic toy set except the puzzle had flashing lights. Each toy in the electronic toy set except the sensory balls made sounds, talked, sang, and/or played music. The toys in the traditional toy set had no electronic features or technological enhancements.

**TABLE 2:** The toy sets.

	Electronic	Traditional
Barn with animals	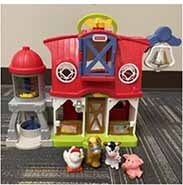	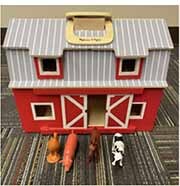
Shape sorter	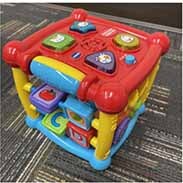	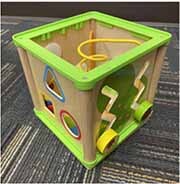
Animal puzzle	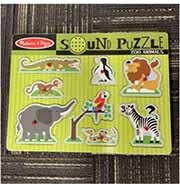	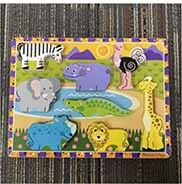
Vehicles	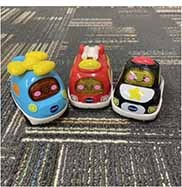	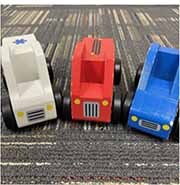
Pull toy dog	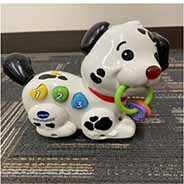	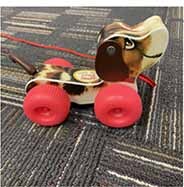
Spiky sensory balls	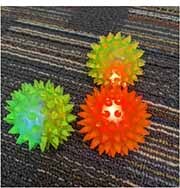	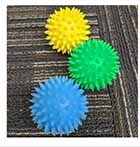

We included a variety of toys in each set to increase the likelihood that each dyad would find one or more toys that interested them. We chose these toys because they are developmentally appropriate for children with a broad range of language and cognitive levels. In addition, the toys are representative of the types of toys commonly available for consumers to purchase online and in stores and have been used in previous studies (Wooldridge and Shapka, 2012; Zosh et al., 2015; Sosa, 2016; Miller et al., 2017). The toy sets were presented in counter-balanced order across participants. In the ASD group, the traditional toy set was provided first to eight parent-child dyads and the electronic toy set was provided first to six dyads. In the TD group, 7 dyads played with the traditional toys first, and 7 dyads played with the electronic toys first.

In the ASD group, the average sample length was 10.26 min (*SD* = 0.40; range = 10.00–11.32) for traditional toy play and 10.50 min (*SD* = 0.96; range = 10.00–13.37) for electronic toy play. In the TD group, the average sample length for traditional toy play was 10.46 min (SD = 0.50; range = 10.0–11.52) and the average sample length for electronic toy play was 10.59 (SD = 0.57; range = 10.00–11.67). Dependent variables were represented as a rate (average count per minute) to account for slight variations in sample length.

### Transcription

Research assistants used Systematic Analysis of Language Transcripts software (SALT; Miller and Iglesias, 2020) to transcribe the play sessions. Each transcriber completed a comprehensive online training program prior to coding independently. The transcription process involved a first pass by a primary transcriber, review and feedback from a secondary transcriber, and final discussion and consensus transcription by the pair. Coders were aware of the toy condition because the toys were visible (and audible, in the case of electronic toys; also see Sosa, 2016). Following standard SALT procedures, utterances were segmented based on communication units (each independent clause and its modifiers). We derived two variables from SALT that represented the quantity and lexical diversity of child spoken language. Variables were represented as a rate (average count per minute) to account for small variations in sample length. Quantity was measured by the number of child utterances per minute. Lexical diversity was measured by the number of different word (NDW) roots per minute that children produced. Only complete and intelligible child utterances were included in these calculations.

To evaluate inter-transcriber agreement, a separate primary and secondary transcriber independently re-transcribed the videos from 16 randomly selected play sessions (four Traditional and four Electronic videos for the TD and ASD groups). We then compared the number of child utterances and the NDW roots derived from each independent transcription. On average, the transcripts differed by two child utterances in the ASD group and by three child utterances in the TD group. On average, the transcripts differed by two different word roots in the ASD group and four word roots in the TD group. Thus, inter-transcriber outcomes for both of the key dependent variables were closely aligned, differing by no more than an average of 2–3 utterances and 3–4 different word roots.

### Analysis Plan

This study involved a within-subject manipulation, wherein each parent-child dyad played with both electronic and traditional toys. Given this within-subject design, as well as the significant differences in language and cognitive skills between the ASD and TD groups, we conducted separate analyses for each group to determine whether the quantity and lexical diversity of children’s spoken language differed by toy type. Some children with ASD produced very little (to no) spoken language. For this reason, we were more interested in which toy type elicited the most child spoken language than in the magnitude of these effects (which would be tested by parametric tests). Given this goal, as well as the relatively modest sample sizes, we analyzed the difference between toy types using Wilcoxon rank-sum tests, the non-parametric analog of a paired-samples *t*-test. We set alpha at 0.05. Because there was a clear prediction and expected direction of effect (i.e., that quantity and lexical diversity of child spoken language would be significantly higher during traditional than electronic toy play), we used 1-tailed tests.

## Results

**FIGURE 1:**
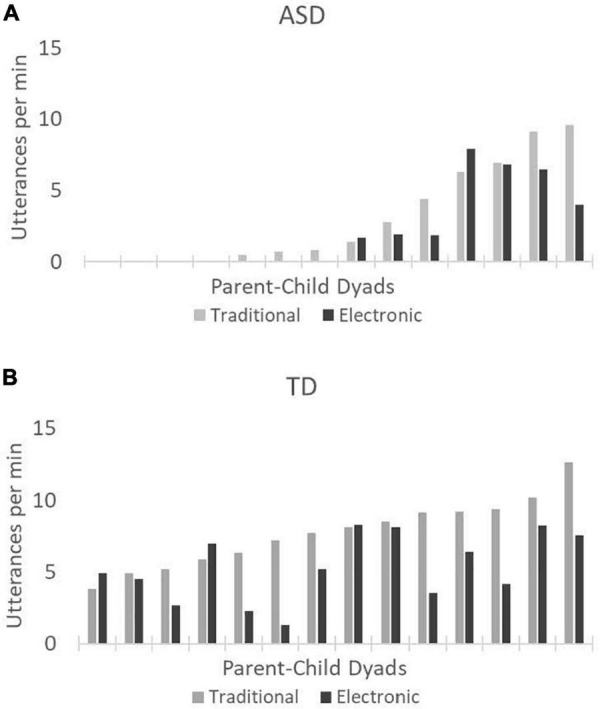
Mean number of utterances per minute produced by the children with autism spectrum disorder (ASD) **(A)** and the children with typical development (TD) **(B)**. Gray bars represent traditional toy play, and black bars represent electronic toy play. Each pair of bars on the *x*-axis represents a single parent-child dyad. Data are presented left to right in order of increasing values for traditional toy play.

The goal of this study was to test the impact of toy type (traditional vs. electronic) on the quantity and lexical diversity of spoken language produced by children with ASD and age-matched children with TD. To examine quantity, we compared the average number of utterances children in each group produced per minute during traditional and electronic toy play (see Figure 1). Children with ASD produced, on average, 3.05 utterances per minute during traditional toy play (median = 1.10, *SD* = 3.55, range = 0–9.61) and 2.21 utterances per minute during electronic toy play (median = 0.90, *SD* = 2.88, range = 0–7.90). Children with TD produced, on average, 7.74 utterances per minute during traditional toy play (median = 7.92, *SD* = 2.37, range = 3.84–12.66) and 5.29 utterances per minute during electronic toy play (median = 5.06, *SD* = 2.34, range = 1.30–8.29). Wilcoxon rank sum tests revealed that the mean number of child utterances per minute was significantly lower during electronic toy play than traditional toy play for both the children with ASD (1-tailed *p* = 0.025) and the children with TD (1-tailed *p* = 0.004).

**FIGURE 2:**
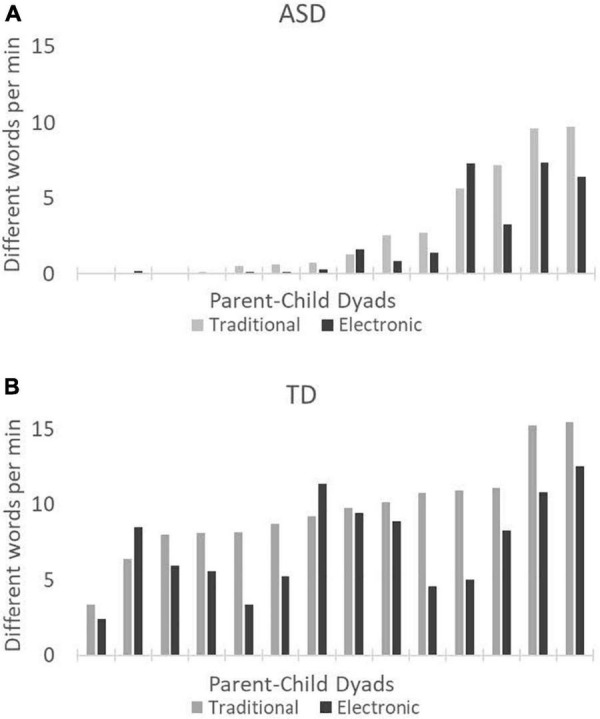
Mean number of different (i.e., unique) words per minute produced by the children with autism spectrum disorder (ASD) **(A)** and the children with typical development (TD) **(B)**. Gray bars represent traditional toy play, and black bars represent the electronic toy play. Each pair of bars on the *x*-axis represents a single parent-child dyad. Data are presented left to right in order of increasing values for traditional toy play.

To examine lexical diversity, we compared the average number of different (i.e., unique) word roots children produced per minute during traditional and electronic toy play (see Figure 2). Children with ASD produced, on average, 2.90 unique words per minute during traditional toy play (median = 1.00, *SD* = 3.61, range = 0–9.75) and 2.06 unique words per minute during electronic toy play (median = 0.56, *SD* = 2.85, range = 0–7.36). Children with TD produced, on average, 9.66 unique words per minute during traditional toy play (median = 9.46, *SD* = 3.14, range = 3.40–15.45) and 7.27 unique words per minute during electronic toy play (median = 7.10, *SD* = 3.21, range = 2.40–12.51). Wilcoxon rank sum tests revealed that the mean number of unique words per minute was significantly lower during electronic toy play than traditional toy play for both the children with ASD (1-tailed *p* = 0.021) and the children with TD (1-tailed *p* = 0.005).

## Discussion

To our knowledge, this study provides the first evidence that electronic toys decrease the quantity and lexical diversity of children’s spoken language, relative to traditional toys. Children with ASD and age-matched children with TD talked significantly less and produced significantly fewer unique words when playing with electronic toys than with traditional toys. Observations of the electronic play sessions indicated that the talking, singing, music, and animal sounds produced by the toys often left little room for children to contribute. The talking, singing, sounds, and flashing lights of the electronic toys dominated the interaction, interrupting children’s utterances and decreasing the space available for parent-child communication. Although electronic toys are often advertised as educational (Levin and Rosenquest, 2001; Healey et al., 2019; Hassinger-Das et al., 2021; Zero to Three, 2021), the current findings add to growing evidence that electronic toys decrease the quality of play interactions between children and their parents (Zosh et al., 2015; Sosa, 2016; Sturman et al., 2022).

In combination with parallel findings in parents (Sturman et al., 2022), these results indicate that electronic toys limit the reciprocal linguistic exchanges between children and parents. This is unfortunate because electronic toys are not a substitute for meaningful communicative exchanges between parents and children. The value of play lies in shared engagement between play partners. When children are engaged in play that encourages linguistic interaction, they have more opportunities to initiate verbal interactions, experiment with grammatical forms, and participate in reciprocal conversational turns. In addition, a reduction in child spoken language limits a parent’s ability to respond to and build on children’s verbal communication, which is an important avenue for language development (Sameroff and Fiese, 2000; Camarata and Yoder, 2002). Though children with TD may be relatively unaffected by these changes, children with ASD are likely to be vulnerable even to seemingly subtle disruptions in parent-child interactions. Electronic toys may compromise the potentially fragile play-based interactions that parents and caregivers create.

Examining the patterns of individual children offers additional insights into the decrease in children’s spoken language during electronic toy play. For example, one child with ASD produced an average of four utterances per minute during electronic toy play and 10 utterances per minute during traditional toy play—a more than twofold increase that yielded approximately 40 utterances (electronic) vs. approximately 100 utterances (traditional) over the full 10-min play samples. In addition, three children with ASD produced only a single utterance during electronic toy play, but produced 5, 7, and 8 utterances, respectively, during traditional toy play. For children in the earliest stages of spoken language development, there is a clinically significant difference between producing 1 utterance in a 10-min play session vs. 5, 7, or 8 utterances. It is important to recognize that the play sessions in the current study lasted 10 min; differences between electronic and traditional toys may be even more dramatic as they accumulate over longer periods of time (Zosh et al., 2015).

Though our primary focus was on children with ASD, it is interesting to note that the age-matched children with TD also produced significantly fewer utterances and used significantly fewer unique words when playing with electronic toys than traditional toys. This was the case even though the children with TD had significantly stronger language skills than the children with ASD, suggesting that electronic toys decrease the quantity and lexical diversity of child spoken language regardless of developmental stage. In other words, the current findings suggest that the quantity and lexical diversity of a child’s spoken language is likely lower during electronic than traditional toy play whether a child produces single words or 5-word utterances. These findings provide developmental continuity with prior findings that infants with TD produce fewer vocalizations and gestures during electronic toy play than traditional toy play (Sosa, 2016; Miller et al., 2017).

Additional research is needed to determine whether electronic toys disrupt play-based language learning opportunities in other ways. The background noise introduced by electronic toys may make it more difficult for children to understand spoken language, especially when it incorporates speech or other rhythmic sounds (Baker and Holding, 1993; Kirkorian et al., 2009; McMillan and Saffran, 2016; Erickson and Newman, 2017; Godwin et al., 2018; McAuley et al., 2020, 2021). In addition, the salient visual features of electronic toys, such as flashing lights, may compete with other relevant aspects of the child’s visual environment (Radesky and Christakis, 2016). Visual salience exerts a strong influence on attention allocation in children with ASD (Sacrey et al., 2014; Venker et al., 2018, 2020). Salient auditory and visual features of electronic toys may decrease the likelihood that children with ASD will engage in joint attention and may cause them to miss important linguistic and social cues (Miller et al., 2017; Healey et al., 2019). These types of effects are important to investigate not only in lab settings, but also in naturalistic contexts, such as homes or classrooms.

An important next step in this line of work is to characterize the beliefs and attitudes of parents of children with ASD regarding toy selection. Parents of young children (without ASD) commonly consider electronic toys an essential teaching tool, with many parents viewing these toys as offering more educational value than themselves (Shah et al., 2018; Healey et al., 2019). Family members seeking to support language development in children with ASD may be particularly susceptible to the claims that electronic toys offer developmental benefits. Clinical practitioners have a responsibility to help parents become informed consumers—for example, by stressing to parents that they, not the toys, are the most important part of play interactions with their child (Wooldridge and Shapka, 2012; Hassinger-Das et al., 2021).

Though the current findings suggest that traditional toy play should be encouraged, it is not necessary (or realistic) to recommend a complete avoidance of electronic toys. Many children enjoy and are highly motivated by electronic toys, and they may be useful when encouraging children (particularly those with ASD) to request or comment on preferred items (Wooldridge and Shapka, 2012). Electronic toys may also facilitate social engagement and shared enjoyment by serving as a source of humor (Bergen et al., 2009). It may be beneficial for parents of children with ASD to make electronic toys available on a limited and purposeful basis, guided by advice from clinical professionals (Healey et al., 2019).

### Limitations and Strengths

One limitation of the current study was the relatively small sample size (*n* = 28 parent-child dyads; *n* = 14 per group). Though small sample sizes limit statistical power, we consider the likelihood of replicating the current results to be high based on the robustness of the findings and their consistency with previous studies (also see Zosh et al., 2015; Sosa, 2016). In addition, the racial and ethnic diversity of the participant sample was limited, which may reduce the generalizability of the results. Another potential limitation is that the ASD and TD groups were matched on chronological age, rather than language or cognitive skills. It may be advantageous for future studies to include language-matched comparison groups. Future work focused on naturalistic contexts is also needed to complement lab-based studies like this one. Future studies may also examine more fine-grained patterns of interaction that unfold over the course of a play session. The current study also had several strengths. Its controlled, within-participants design allowed each parent-child dyad to serve as their own control, thereby removing potential confounds introduced by the unique interaction styles of individual dyads (Sosa, 2016). The toy sets were closely matched and included a variety of developmentally appropriate toys. Another strength was the rigorous manual transcription process, which involved a primary and secondary transcriber and consensus coding process.

## Conclusion

The current findings indicate that electronic toys reduce the quantity and lexical diversity of spoken language produced by children with ASD and age-matched children with TD, thereby undermining play-based language learning opportunities. These findings add to growing empirical evidence that expensive, technologically enhanced toys are not necessary for young children’s learning—and, in fact, may be detrimental. Play-based interventions for children with ASD may be most effective when they incorporate traditional toys, rather than electronic toys. These findings also make it possible for clinical practitioners to provide evidence-based recommendations about toy selection to families of children with ASD. Parents should be assured that no toy can take the place of a sensitive, engaged, responsive play partner. Well-targeted, sensitive recommendations will take individual parent and caregiver beliefs into account to ensure practitioners demonstrate respect for parents’ efforts to help their children.

## Data Availability Statement

The raw data supporting the conclusions of this article will be made available by the authors, without undue reservation.

## Ethics Statement

The studies involving human participants were reviewed and approved by the Michigan State University IRB. Written informed consent to participate in this study was provided by the participants’ legal guardian/next of kin.

## Author Contributions

CV conceptualized the study, conducted the statistical analyses, and obtained funding for data collection. Both authors formulated the research question and helped write the manuscript.

## Conflict of Interest

The authors declare that the research was conducted in the absence of any commercial or financial relationships that could be construed as a potential conflict of interest.

## Publisher’s Note

All claims expressed in this article are solely those of the authors and do not necessarily represent those of their affiliated organizations, or those of the publisher, the editors and the reviewers. Any product that may be evaluated in this article, or claim that may be made by its manufacturer, is not guaranteed or endorsed by the publisher.
